# A tyrosine kinase inhibitor-induced interferon response positively associates with clinical response in EGFR-mutant lung cancer

**DOI:** 10.1038/s41698-021-00181-4

**Published:** 2021-05-17

**Authors:** Natalia J. Gurule, Caroline E. McCoach, Trista K. Hinz, Daniel T. Merrick, Adriaan Van Bokhoven, Jihye Kim, Tejas Patil, Jacob Calhoun, Raphael A. Nemenoff, Aik Choon Tan, Robert C. Doebele, Lynn E. Heasley

**Affiliations:** 1 Department of Craniofacial Biology, University of Colorado Anschutz Medical Campus, Aurora, CO USA; 2 Department of Medicine and Helen Diller Family Comprehensive Cancer Center, University of California, San Francisco, CA USA; 3 Department of Pathology, University of Colorado Anschutz Medical Campus, Aurora, CO USA; 4 Department of Medicine, University of Colorado Anschutz Medical Campus, Aurora, CO USA; 5 Moffitt Cancer Center, Tampa, FL USA; 6 Eastern Colorado VA Healthcare System, Rocky Mountain Regional VA Medical Center, Aurora, CO USA

**Keywords:** Non-small-cell lung cancer, Oncogenes, Inflammation, Translational research

## Abstract

Tyrosine kinase inhibitors (TKIs) targeting EGFR-mutant lung cancers promote a range of tumor regression responses to yield variable residual disease, a likely incubator for acquired resistance. Herein, rapid transcriptional responses induced by TKIs early in treatment that associate with the range of patient responses were explored. RNAseq was performed on EGFR mutant cell lines treated in vitro with osimertinib and on tumor biopsies of eight EGFR mutant lung cancer patients before and after 2 weeks of TKI treatment. Data were evaluated for gene expression programs altered upon TKI treatment. Chemokine and cytokine expression were measured by ELISA and quantitative RT-PCR. IκB Kinase (IKK) and JAK-STAT pathway dependence was tested with pharmacologic and molecular inhibitors. Tumor sections were stained for the T-cell marker CD3. Osimertinib stimulated dynamic, yet wide-ranging interferon (IFN) program regulation in EGFR mutant cell lines. IL6 and CXCL10 induction varied markedly among the EGFR mutant cell lines and was sensitive to IKK and JAK-STAT inhibitors. Analysis of matched patient biopsy pairs revealed marked, yet varied enrichment of IFN transcriptional programs, effector immune cell signatures and T-cell content in treated tumors that positively correlated with time to progression in the patients. EGFR-specific TKIs induce wide-ranging IFN response program activation originating within the cancer cell. The strong association of IFN program induction and duration of clinical response indicates that the TKI-induced IFN program instructs variable recruitment and participation of immune cells in the overall therapeutic response.

## Introduction

Lung cancers bearing the oncogenic receptor tyrosine kinases (RTKs), EGFR, ALK, and ROS1, are amenable to precision oncology treatments with distinct tyrosine kinase inhibitors (TKIs) that provide superior tumor regression, progression-free survival, overall survival, and quality of life compared to standard chemotherapy^1–6^. While tumor shrinkage can be profound, TKIs fail to eliminate 100% of cancer cells, yielding residual disease which functions as an incubator from which lethal drug-resistant cancers ultimately emerge^7^. Moreover, objective responses to TKIs vary widely among oncogene-defined patient cohorts as illustrated by the prototypic waterfall plot. We have previously noted the existence of an association between the depth of response and progression-free and overall survival in ALK + patients^8^. The important biology underlying the variable depth of response must be approached with deep analysis of residual disease early during treatment, not late at treatment failure. The results may highlight important mechanisms and pathways for rational targeting in combination with TKIs to increase clinical benefit.

Tumor heterogeneity represents an obvious explanation for the range of therapeutic responses observed in an oncogene-defined patient cohort. Tumor heterogeneity is regulated by many factors, both extrinsic and intrinsic to the tumor cell where sources of intrinsic heterogeneity include clonal evolution by which stochastic accumulation of mutations leads to the development of distinct genetic subclones^9,10^. The tumor microenvironment (TME) is a source of extrinsic heterogeneity as it is comprised of multiple cellular components including fibroblasts, extracellular matrix factors, lymphatic vasculature, as well as diverse infiltrating immune cell types eliciting both pro- and anti-tumorigenic capabilities^11^. Both intrinsic and extrinsic factors contribute to different functional properties of tumors, including resistance and response to therapy. Still, most decisions regarding lung cancer treatment are based on knowledge of a single oncogenic driver without consideration for the functional consequences of treatment with oncogene-specific drugs beyond targeting a growth vulnerability in a tumor cell.

Mechanisms of acquired resistance to oncogene specific TKIs have been identified in tumor specimens obtained at treatment failure and include alterations in the drug target through development of resistance mutations, activation of bypass signaling pathways, and phenotype switching^12–20^. Notably, deployment of serial monotherapies designed to target these emergent resistance pathways have not yet transformed lung cancer from a fatal disease into a chronic or curable one^7,14^. By comparison, little information is available regarding early TKI-induced biochemical and cellular events activated in residual disease. To this end, we have characterized acute EGFR-specific TKI-induced transcriptional reprogramming in vitro with EGFR mutant lung cancer cell lines as well as matched pairs of pre- and on-treatment biopsies of primary EGFR mutant lung tumors. The results reveal a general induction, albeit remarkably varied among cell lines and patients, of inflammation-related transcriptional pathways predicted to regulate host immune interactions. The positive association of the degree of an interferon response program induction with the duration of patient response to EGFR-specific TKI supports a hypothesis that this paracrine signaling response is a significant, yet variable contributor to the overall therapeutic response. Identifying impactful ways to broaden this TKI-stimulated transcriptional program in lung tumors may provide a path to more durable clinical response in EGFR mutant lung cancers.

## Results

### RNAseq analysis reveals broad transcriptional reprogramming and induction of an interferon response upon treatment with EGFR TKI osimertinib

A panel of six human lung cancer cell lines bearing activating mutations in EGFR was tested for growth sensitivity to the 3rd generation TKI, osimertinib, and representative dose–response curves are shown in Supplementary Fig. S1. The calculated IC_50_ values for the panel of cell lines ranged from 2 nM to 9 nM for osimertinib, establishing that the cell lines display similar and clinically relevant growth sensitivity to EGFR inhibition. To determine the effect of osimertinib treatment on cell viability, PC9, H1650, HCC4006, and H1975 cells were treated with 300 nM osimertinib for 3 days and PARP cleavage was measured by western blot. PC9 cells are the only cell line in which treatment with osimertinib induces cell death as assessed by cleaved PARP (Supplementary Fig. 1C), while the remaining cell lines exhibit a cytostatic effect.

**Fig. 1:**
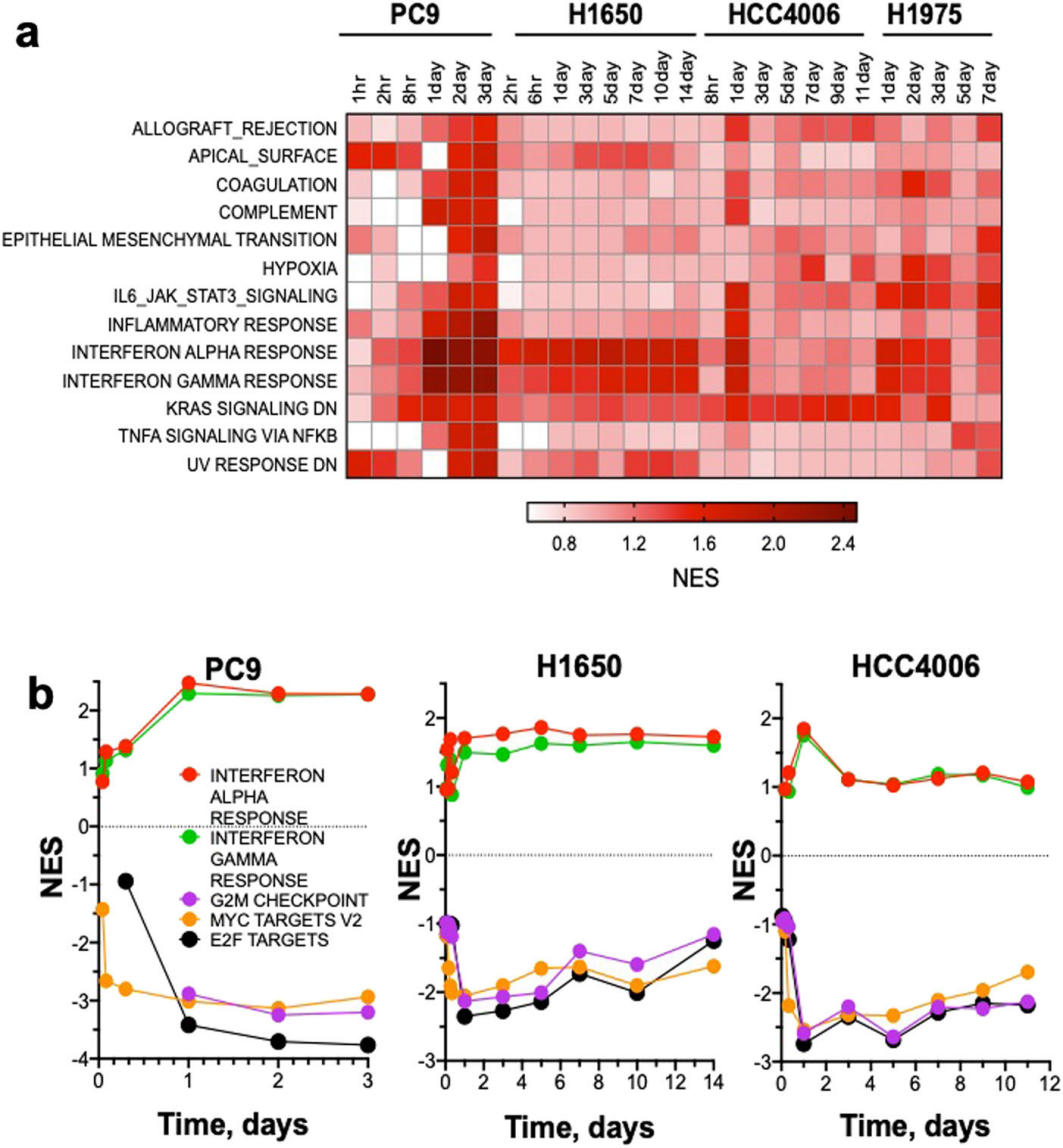
Inflammation-associated gene sets including IFN response genes are broadly but variably induced by osimertinib treatment. RNAseq was performed on EGFR mutant lung cancer cell lines PC9, H1650, HCC4006, and H1975 treated in vitro with 300 nM osimertinib or DMSO control for the indicated times. The data are from one distinct sample per time point. **a** Gene Set Enrichment Analysis (GSEA) was performed using the Hallmark MSigDB gene sets and the Normalized Enrichment Scores (NES) for the high-ranking gene sets are indicated for the osimertinib-treated sample relative to DMSO control. **b** For PC9, H1650 and HCC4006 cells, positive NES for IFNα and IFNγ gene sets are graphed in contrast to negative enrichment scores for cell cycle associated gene sets G2M checkpoint, MYC Targets V2, and E2F Targets.

RNAseq was performed on total RNA purified from EGFR mutant lung cancer cell lines, H1650, HCC4006, and H1975 treated in vitro with 300 nM osimertinib or DMSO diluent over a time course ranging from 1 h to 14 days. PC9 cells underwent extensive cell death in response to osimertinib such that the longest time point studied was 3 days. Because osimertinib exerted a cytostatic effect on H1650, HCC4006, and H1975 cells, longer times of incubation could be investigated. To search for transcriptional programs regulated by osimertinib, Gene Set Enrichment Analysis (GSEA)^21^ was performed using the Hallmark Gene sets from the Molecular Signature Database (MSigDB)^22^. These gene sets represent well-defined biological states and processes which can be used as an unbiased approach to identify function associated with changes in gene expression. The resulting Hallmark gene sets were filtered based on nominal (NOM) *p* value <0.05 and Normalized Enrichment Scores (NES) were used to identify top-ranked gene sets for each time point across the four cell lines in the osimertinib treated versus the DMSO control. Enrichment of multiple Hallmark gene sets in response to osimertinib was observed, many of which are related to inflammation (Fig. 1a and Supplementary Table 1). Notably, the Interferon Alpha Response (IFNα) and Interferon Gamma Response (IFNγ) Hallmark pathways were the top-ranking gene sets among the four cell lines, although each cell line displayed distinct kinetics for peak NES score (Fig. 1a, b). The magnitude of enrichment for both IFNα and IFNγ response was the largest in PC9 cells, with a peak NES score of 2.47 and 2.29, respectively, occurring after 1 day of osimertinib treatment. Similarly, IFNα and IFNγ programs were rapidly induced in H1650 cells with peak enrichment after 5 days of TKI treatment with a NES score of 1.86 for IFNα response and 1.62 for IFNγ response. Finally, enrichment occurred rapidly, but transiently after 1 day of osimertinib treatment in HCC4006 cells, with peak NES scores of IFNα = 1.84 and IFNγ = 1.75. The IFNα and IFNγ response gene sets are induced to maximal enrichment of 1.7 and 1.6 respectively after 1 day of EGFR treatment in H1975 cells (Fig. 1a and data not shown). These data reveal that an IFN response is rapidly induced upon treatment with osimertinib in EGFR mutant lung cancer cell lines, albeit with distinct magnitude and kinetics.

Hallmark gene sets that exhibited negative enrichment in the osimertinib-treated cell lines were also identified and included the growth-associated gene sets, G2M Checkpoint, MYC Targets, and E2F Targets (Fig. 1b). These three gene sets were rapidly downregulated at the earliest time point in the time course of TKI treatment and subsequently sustained at longer treatment times. The findings that the magnitude and kinetics of negative enrichment of the growth-associated gene sets were similar across all cell lines (Fig. 1b) while positive enrichment varied over the same time course indicates that induction of the interferon response is not simply a result of growth inhibition by osimertinib.

To assess TKI-induced transcriptional reprogramming in vivo, H1650 and PC9 cells were implanted in the flanks of nu/nu mice and tumors were allowed to establish. Tumor-bearing mice were treated by daily oral gavage with 5 mg/kg osimertinib or diluent control for 2 weeks. Both PC9 and H1650 flank xenografts exhibited tumor shrinkage in response to osimertinib treatment (Supplementary Fig. 2A), although the degree of response was deeper in the PC9 tumors than the H1650 tumors. RNA from H1650 and PC9 xenografts treated for 2 weeks with osimertinib or diluent control were submitted to RNAseq and GSEA was performed. NES scores from the Hallmark gene sets were ranked for both osimertinib-induced and inhibited pathways and presented in Supplemental Fig. S2B. Similar to the in vitro results, both the IFNγ and IFNα response gene sets were among the highest-ranked pathways in the osimertinib-treated tumors. In addition, many other inflammation-associated gene sets such as Allograft Rejection and Complement were enriched upon osimertinib treatment. Gene sets with negative enrichment in the EGFR TKI-treated group were mainly gene sets with growth-associated genes such as G2M Checkpoint, E2F Targets, and MYC Targets, all of which indicate the growth inhibitory effect of osimertinib on these xenografts. These findings demonstrate that the EGFR TKI-induced IFN response is observed both in vitro and in xenograft models treated systemically with osimertinib.

### Osimertinib-regulated interferon response genes include anti-viral effectors and diverse chemokines, cytokines, and interleukins with pro-tumorigenic and anti-tumorigenic functions

The GSEA Hallmark IFNα and IFNγ response gene sets are comprised of 297 distinct genes known to be regulated by type I or type II IFNs^23–25^. Many of the genes are involved in innate immune function including anti-viral effectors, but also include diverse chemokines and cytokines, antigen presentation machinery and transcription factors known to drive many of these responses (Supplementary Fig. 3A). Interferon gene family members were, in general, not expressed and none exhibited increased expression after osimertinib treatment in any of the lung cancer cell lines, indicating that this program is not driven by autocrine interferon signaling. As assessed by mRNA expression of the cell proliferation markers, MKI67 and PCNA, cell growth was promptly and strongly reduced in the four cell lines with evidence of modest re-expression following 1–2 weeks of osimertinib treatment (Supplementary Fig. 3A). Thus, consistent with the similar efficacy of osimertinib for inhibiting growth (Supplementary Fig. 1), the kinetics and magnitude of inhibition of proliferation-related gene expression were similar among the EGFR mutant cell lines (Fig. 1a, b, Supplementary Fig. 3A). This finding is further supported by evidence of deceased activity of the growth-associated GSEA gene sets G2M checkpoint, MYC targets, and E2F targets observed in Fig. 1b.

In contrast to inhibition of proliferation-related genes, induction of distinct IFN response genes varied markedly among the four cell lines (Supplementary Fig. 3A). In general, HCC4006 exhibited transient and modest induction of the IFN program following osimertinib treatment that peaked at 1–3 days and then returned to baseline levels observed in DMSO-treated cells (Fig. 1b, Supplementary Fig. 3A). H1650 cells exhibited a slower onset in IFN pathway induction, requiring ~1 week of osimertinib treatment to achieve maximum activation of gene expression that was maintained for the duration of the experiment. Notably, H1650 cells exhibited profound increases, relative to the other cell lines, in expression of genes with anti-viral function such as IFIT1, IFIT3, MX1, and MX2 (Supplementary Fig. 3A). PC9 cells exhibited rapid induction of IFN pathway genes within 1 day of osimertinib treatment and expression was maintained for the 3-day time course. H1975 cells exhibited a response pattern intermediate to the other cell lines with modest, but more sustained activation than HCC4006. The levels of STAT1 and STAT2 mRNAs mirrored the kinetic trajectories observed for other IFN Hallmark genes in the osimertinib-treated lung cancer cell lines but were especially marked in H1650 cells (Supplementary Fig. 3A). The genes encoding these transcription factors are IFN regulated and also central inducers of many of the IFN-responsive genes^26,27^. STAT2 appears to be more robustly regulated than STAT1 at the mRNA level. While NFκB family genes, RELA and RELB, were more constitutively expressed, the IκBα gene, NFKBIA, represents a NFκB pathway target and was upregulated following osimertinib treatment in HCC4006, H1975, and PC9 cells (Supplementary Fig. 3A). Finally, IFN Hallmark Pathway genes involved in antigen presentation (B2M, HLA-E, HLA-DMA) were induced following osimertinib treatment with kinetics characteristic of the distinct cell lines (Supplementary Fig. 3A). Osimertinib-induced IFN response gene expression changes were validated by qRT-PCR analysis in H1650 and HCC4006 cells and extended to two additional EGFR mutant, osimertinib-sensitive cell lines, HCC827 and HCC2935 (Supplementary Fig. 4). Expression of an anti-viral gene (IFIT1) as well as STAT1 and STAT2 were rapidly induced at the mRNA level within 2–3 days of osimertinib treatment and generally sustained for 7 days (Supplementary Fig. 4). Finally, immunoblot analysis verified that protein levels of IFIT1, MX2, and STAT1 were increased following osimertinib treatment in a cell line-specific manner similar to induction of mRNA (Supplementary Fig. 3B).

Within the shared IFNα response and IFNγ response Hallmark gene sets, a significant fraction encode chemokines, cytokines, and interleukins with both anti- and pro-tumorigenic roles in communicating with diverse immune cells in the TME^11^. For example, CXCL10 is a member of the CXCL9/10/11 family of chemokines that are ligands for CXCR3, a receptor that is expressed on B and T cells as well as NK cells, dendritic cells and macrophages. The production of CXCL10 by cancer cells acts as a chemoattractant for anti-tumorigenic immune cell subsets such as effector T cells^28^. By contrast, IL6 is a member of a family of cytokines with established roles in acute phase response, inflammation, hematopoiesis, and cancer progression^29,30^. Cancer cell-intrinsic functions of IL6 include promoting proliferation and survival thereby functioning as a bypass pathway in the setting of EGFR-specific TKIs^31^. Cancer cell-extrinsic functions include promotion of angiogenesis and immune evasion.

In addition to CXCL10 and IL6, several chemokines, cytokines, and interleukins identified as osimertinib-regulated in the RNAseq data are presented in Supplementary Fig. S3A and validated by quantitative RT-PCR (Supplementary Fig. 5). Gene expression was validated by qRT-PCR in the cell lines that were submitted to RNAseq (PC9, H1650, H1975, and HCC4006) and expanded to two additional cell lines, HCC827 and HCC2935. Notably, great diversity was observed in the chemokine/cytokine genes that were regulated in distinct cell lines, although when a gene was regulated, it occurred with the kinetics typical of other IFN response genes in that lung cancer cell line. H1650 cells exhibited marked osimertinib-induced expression of CXCL10, CCL5, and CCL28 and modest induction of IL32 mRNA. By contrast, CXCL1, 2, and 8 mRNAs were markedly induced by osimertinib in HCC4006, PC9, and H1975, but not in H1650 cells (Supplementary Fig. 5). Specific chemokines like CCL2 were only significantly expressed and regulated by osimertinib in HCC4006 cells while CCL5 regulation was a dominant event in H1650 cells and more modest in H1975 and HCC2935 (Supplementary Figs. 3A and 5). The pro-tumorigenic cytokine, TGFB2, was induced by osimertinib at the mRNA level in all of the cell lines tested except for H1650 (Supplementary Figs. 3A and 5). Notably, TGFB2 is not included in the IFNA or IFNG Hallmark pathways but is a conserved gene on multiple YAP-pathway signatures^32^. In addition to IFN family members which were not regulated by osimertinib treatment, TNFα was not expressed in any of the EGFR mutant cell lines and IL1α/IL1β were either unregulated or inhibited in expression (data not shown).

**Fig. 2:**
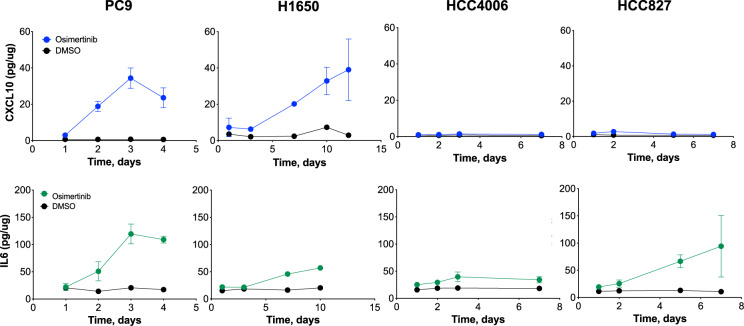
Distinct patterns of CXCL10 and IL6 induction in EGFR mutant lung cancer cell lines treated with osimertinib. Media from PC9, H1650, HCC4006, and HCC827 cells treated with 300 nM osimertinib or DMSO control for the indicated time points was collected and submitted to ELISA measurements of CXCL10 and IL6 protein. The data are the mean and SEM of three independent experiments.

Regulation of CXCL10 and IL6 protein were measured by ELISA in growth medium from control and osimertinib-treated PC9, H1650, HCC4006, and HCC827 cells (Fig. 2). CXCL10 protein levels coincided closely in magnitude and kinetics with mRNA levels (Supplementary Figs. 3A and 5). By contrast, IL6 protein secretion did not correlate as closely with mRNA levels. For example, IL6 mRNA is more strongly induced by osimertinib in HCC4006 cells relative to PC9 cells (Supplementary Figs. 3A and 5), but protein expression is higher in PC9 cells (Fig. 2). To broaden the measurement of chemokines and cytokines produced by the lung cancer cells in response to osimertinib treatment, growth medium from control and TKI-treated cells were analyzed with a multiplexed Luminex assay (Supplementary Fig. 6). The analysis validated the CXCL10 and IL6 ELISA results (Fig. 2), the lack of induction of CXCL1 in H1650 cells relative to the other cell lines (Supplementary Fig. 3A), and the unique induction of CCL5 in H1650 cells (Supplementary Figs. 3A and 5). These findings reveal the expression of a complex constellation of chemokines, cytokines, and interleukins that is distinct among EGFR mutant lung cancer cell lines treated with osimertinib. Considering their diverse cellular targets, the predicted in vivo function on the tumor immune microenvironment is likely to be equally varied.

### Signal pathway dependencies for EGFR TKI-induced chemokine and cytokine expression

**Fig. 3:**
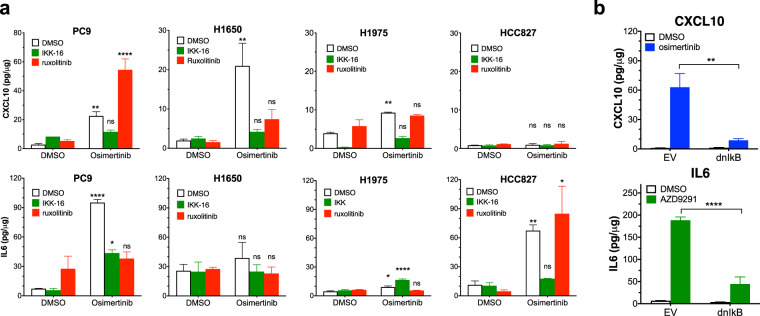
Osimertinib-induced expression of CXCL10 and IL6 is NFkB and JAK/STAT pathway dependent. **a** PC9, H1650, H1975, and HCC827 cells were treated with DMSO or 300 nM osimertinib alone and in combination with 500 nM IKK16 or 500 nM ruxolitinib. All cell lines were treated for 7 days except PC9 cells which were treated for 3 days. Secreted CXCL10 and IL6 were measured by ELISA. The data are from three independent experiments and were submitted to one-way ANOVA and statistical significance of the osimertinib-treated samples relative to their DMSO controls is shown. **b** PC9 cells expressing the dnIκB protein or empty vector as a control were treated with 300 nM osimertinib or DMSO control for 3 days and CXCL10 and IL6 protein secretion into the media was measured by ELISA. The data are the mean and SEM of three independent experiments and presented as pg/μg protein.

The GSEA findings demonstrate a robust increase in anti- and pro-tumorigenic factors such as CXCL10 and IL6 in response to EGFR TKI. TKI-induced expression of STAT1/STAT2 and NFKBIA, an NFκB target gene^33,34^, suggest both signal pathways may mediate the overall innate immune response. Also, both the NFκB and JAK-STAT pathways are known to contribute to the regulation of CXCL10 and IL6 expression^35^. IκB Kinase (IKK) phosphorylates IκB molecules leading to their ubiquitinoylation and degradation, thereby permitting translocation of NFκB to the nucleus and transcriptional activation of target genes. The RNAseq findings indicated TKI increased expression of STAT1 and especially, STAT2, in the EGFR mutant cell lines (Supplementary Fig. S3A). To test the role of the NFκB pathway, IKK16, an IKKα/β inhibitor was deployed while ruxolitinib, a JAK1/JAK2 inhibitor, was used to test the requirement for the JAK/STAT pathway. EGFR mutant cell lines were treated with DMSO control, 300 nM osimertinib alone and in combination with either IKK16 or ruxolitinib and secreted CXCL10 and IL6 were measured by ELISA (Fig. 3a). Treatment time points for each cell line were selected based on maximal protein expression from the osimertinib treatment time courses (Fig. 2). In PC9 and H1975 cells, treatment with IKK16 significantly reduced osimertinib-induced CXCL10 protein and, in PC9 cells, also IL6 protein (Fig. 3a) while ruxolitinib was without significant effect. IL6 is not induced by osimertinib in H1650 cells, but both IKK16 and ruxolitinib inhibited osimertinib-stimulated CXCL10 expression to similar degrees (Fig. 3a). Consistent with Supplementary Fig. S6, CXCL10 protein is not induced in HCC827 cells (Fig. 3a), but osimertinib-stimulated expression of IL6 was sensitive to IKK16 and not ruxolitinib. It is notable that the ruxolitinib sensitivity of CXCL10 induction is limited to H1650 cells where osimertinib-stimulated expression of STAT1/2 is more dominant (Supplementary Figs. 3A and 4). Because IKKε has been identified as a downstream signaling component in TKI-induced interferon signaling^36^, we tested the effect of three distinct TBK1/IKKε inhibitors (BX795, MRT-67307, and amlexanox) on osimertinib-stimulated CXCL10 secretion from H1650 and PC9 cells. None of these TBK1/IKKε inhibitors reduced osimertinib-stimulated CXCL10 expression (data not shown). Thus, in our hands, TKI-induced signaling through an IKKα/β dependent-pathway is a dominant regulator of the osimertinib-stimulated IFN response. To validate the pharmacological findings with IKK16, a dominant-negative, non-phosphorylatable IκB construct (dnIκB) was stably expressed in PC9 cells. Attempts to express dnIκB in H1650 cells failed, indicating an essential role for NFκB in proliferation and survival in this cell line (data not shown). Consistent with the effect of IKK16, PC9 cells expressing dnIκB exhibited markedly reduced induction of CXCL10 and IL6 protein following osimertinib treatment relative to cells transduced with an empty vector (Fig. 3b). The findings demonstrate requirements for both NFκB and in H1650 cells, JAK/STAT pathways in regulation of CXCL10 and IL6 by osimertinib.

### RNAseq analysis of lung tumor biopsies obtained from patients treated with EGFR TKI reveals induction of multiple inflammation-related programs

**Fig. 4:**
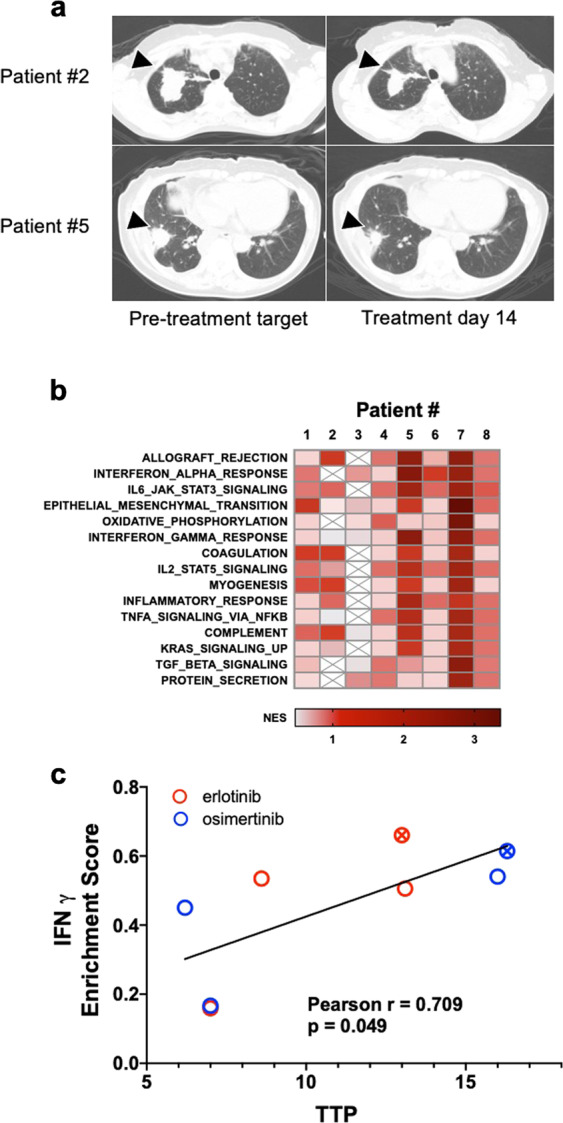
Enrichment of interferon response and inflammation-associated gene sets correlates with progression free survival in human lung tumor biopsies. Lung tumor biopsy specimens from eight EGFR mutant lung cancer patients were collected prior to treatment (baseline) and after 2 weeks of treatment with an EGFR TKI (re-biopsy). **a** Representative CT images of two patients acquired pre-treatment and at re-biopsy are shown. The arrowheads indicate the lesion that underwent biopsy prior to and on treatment with EGFR TKIs. **b** Following RNAseq analysis of RNA purified from the biopsies, GSEA was performed using the Hallmark MSigDB gene sets. The mean Normalized Enrichment Scores (NES) for each Hallmark across the 8 biopsy pairs were used to rank the gene sets and the top 15 are presented as a heatmap (the full analysis is provided in Supplementary Table S2). The data are from one distinct sample per biopsy. **c** Pearson correlation analysis was used to compare the enrichment scores from the 50 Hallmark gene sets with the TTP for the 8 patients. The full analysis is presented in Supplementary Table S3. The patient biopsies from the two EGFR-L858R tumors are indicated with a circle containing an “x”.

The in vitro and xenograft studies indicate that osimertinib induced an IFN response that exhibits variation in both kinetics and magnitude among distinct EGFR mutant lung cancer cell lines. To extend these findings, early transcriptional reprogramming stimulated by therapeutic TKIs in patient-derived biopsies of EGFR mutant lung cancers was performed. Lung tumor biopsies were collected under informed consent prior to initiation of treatment (baseline) and after ~2 weeks (10 days to 2 months) of treatment with an EGFR TKI (re-biopsy). The rationale for timing of the on-treatment specimen was that it matched the time interrogated in the cell line models (both in vitro and in vivo) and increased the rate of successful re-biopsy due to sufficient residual tumor tissue. Fig. 4a shows representative CT images of lung tumors prior to and after 2 weeks of EGFR TKI treatment. A total of eight matched biopsy pairs were obtained from patients bearing EGFR exon 19 deletions (*n* = 6) and L858R mutations (*n* = 2), and the therapeutic TKI deployed and time to progression (TPP) for each patient are tabulated (Table 1). The TTP for the eight patients ranged from 6.2–16.3 months. Co-occurring mutations in TP53, PIK3CA, and RAF1 are indicated (Table 1) and while the number of samples is insufficient to test statistical significance, no obvious association of TTP with co-occurring TP53 or PIK3CA mutations or specific therapeutic TKI deployed was observed.

**Table 1:** EGFR mutant lung cancer patients diagnosed at the University of Colorado Hospital and the University of California San Francisco were consented to an IRB-approved protocol to obtain tumor biopsies before and after 2 weeks of treatment with the indicated TKI.

Clinical characteristics of EGFR mutant lung cancer patients							
Patient #	EGFR mutation	TP53	Co-mutations	EGFR TKI	Smoking status	Time to progression	Time on treatment
1	EGFR Exon19del	mut	PIK3CA	Osimertinib	Former	6.2 months	16 days
2	EGFR Exon19del	wt	PIK3CA	Erlotinib	Never	7 months	14 days
3	EGFR Exon19del	mut	RAF1	Osimertinib	Never	7 months	14 days
4	EGFR Exon19del	wt		Erlotinib	Never	8.6 months	10 days
5	EGFR-L858R	mut	N/A	Erlotinib	Never	13 months	14 days
6	EGFR Exon19del	wt		Erlotinib	Never	13.1 months	76 days
7	EGFR Exon19del	mut	APC	Osimertinib	Never	16 months	14 days
8	EGFR-L858R	mut	PIK3CA	Osimertinib	Never	16.3 months	24 days

The specific EGFR mutation, TP53 mutation status, notable co-mutations, smoking status, time to treatment progression, and time on treatment prior to rebiopsy.

To interrogate early transcriptional changes in response to treatment with EGFR TKIs, RNAseq was performed on the eight matched biopsy specimens. The resulting gene expression data were submitted to GSEA using the MSigDB Hallmark gene sets as previously described for the EGFR mutant cell lines treated with osimertinib in vitro (Fig. 1) and in vivo (Supplementary Fig. 2B). There were multiple overlapping enriched gene sets found when the in vitro RNAseq data were compared to the human biopsy data. The common enriched gene sets include multiple immune and inflammation-related Hallmark Pathway gene sets (Fig. 4b and Supplementary Table 2). Immune-related programs dominated the highest-ranking Hallmarks and included Allograft Rejection, IFNA Response, IL6-JAK-STAT3 Signaling, IFNG Response, and IL2-STAT5 Signaling in the top ten pathways. Epithelial-Mesenchymal Transition was also highly ranked. Thus, the transcriptional responses observed in the pre- and on-treatment biopsies are similar to the RNAseq data obtained from cell line models and supports a hypothesis that treatment with EGFR TKI induces inflammatory and IFN responses in patient lung tumors that derives from direct actions of the TKIs on the cancer cells.

### Interferon gamma response pathway enrichment score correlates with time to progression in lung cancer patients treated with EGFR TKIs

Pearson correlation analysis was performed to compare in an unbiased manner the enrichment scores for the 50 Hallmark gene sets and the TTP for the set of patients. The full results of that analysis are presented in Supplementary Table S3 and indicate that only the IFNγ Hallmark response (Fig. 4c) reached statistical significance (*p* < 0.05). However, consistent with the fact that there are many shared genes and functions within the Hallmark pathways related to inflammation, IL6-JAK-STAT3 Signaling, Allograft Rejection, and IFNα Response were also correlated, but with *p* values slightly greater than 0.05 (Supplementary Table S3). As reinforced by the graphical presentation in Fig. 4c, the TTP is not related to the specific TKI deployed in the patients (erlotinib or osimertinib) or the specific EGFR mutation (L858R vs. exon19del). These data demonstrate that patient lung tumors exhibiting a greater magnitude of EGFR TKI-induced IFN responses experience longer times to progression.

**Fig. 5:**
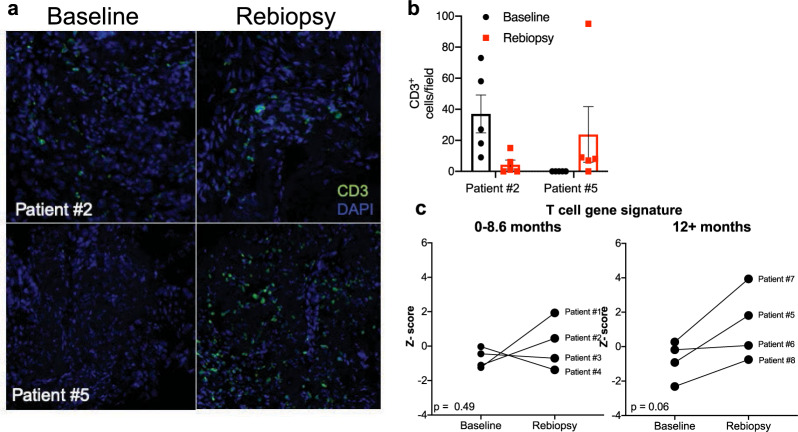
T-cell content upon treatment with EGFR TKI in human lung tumor biopsies. **a** Single representative images of 2 matched lung tumor biopsies submitted to immunofluorescence staining for CD3 is shown. **b** Five fields per biopsy were counted and the individual determinations and their mean are plotted. **c** An immune cell signature developed by Bindea et al.^37^ to deconvolute bulk gene expression data was used to infer T-cell content in the RNAseq data. *Z* scores for signatures predicting T cells were binned by TTP of 0–8.6 months or >12 months. Matched *Z* scores for each patient are graphed at baseline and upon re-biopsy and analyzed by paired *t*-test. The *p* values for the TTP of 0–8.6 months and >12 months are 0.49 and 0.06, respectively.

Replicate pre- and on-treatment biopsies were available from patient #2 and #5 and were submitted to immunofluorescence staining for the T-cell marker, CD3. Single representative images for each patient are displayed in Fig. 5a. Of note, patient #2 progressed in 7 months and patient #5 progressed in 13 months. Following staining, CD3 positive cells were counted in five independent fields per tumor biopsy and the individual values and the average is plotted in Fig. 5b. In patient #2, the tumor was basally inflamed before treatment, and CD3+ T cells decreased after 2 weeks of treatment (Fig. 5b). By contrast, patient #5 exhibited low T-cell counts at baseline and CD3 positive T cells increased after 2 weeks of treatment. A gene signature developed by Bindea et al. deconvolutes complex cell mixtures from bulk RNAseq data to predict immune cell content in a sample^37^. The gene signatures were applied to the patient biopsy RNAseq data and Z scores calculated. The median and mean of the TPP among patients were 10.8 and 10.9 months, respectively. Based on this statistical split in the data, patients were binned based on the duration of response (TPP of 0–8 month or >12 months) and the baseline T-cell *Z*-score was compared to the corresponding re-biopsy *Z*-score. Biopsy pairs from patients exhibiting a TTP in the range of 0–8 months showed increased T-cell scores in two on-treatment biopsies and decreased scores in the other two (Fig. 5c). A paired *t*-test analysis yielded a *p* value of 0.49. By contrast, T-cell signature *Z* scores from biopsy pairs where the patients experienced a TTP of 12 months or greater were increased in 3 of the 4 on-treatment biopsies and the fourth showed little or no change. Paired *t*-test demonstrated a trend toward significance (*p* = 0.06, Fig. 5c). In summary, the data demonstrate a statistically significant association with the IFNγ Hallmark signature and increased TTP. Moreover, the findings support increased T-cell content in on-treatment biopsy specimens from patients experiencing longer TTP. Taken together, these data suggest that EGFR TKIs induce a tumor cell-intrinsic IFN response that communicates with the TME to increase T-cell infiltration that contributes to therapeutic response.

## Discussion

Herein, we demonstrate that treatment of EGFR addicted lung tumor cells with EGFR-specific TKIs results in induction of an interferon response program that includes multiple chemokines and cytokines as well as antigen presentation machinery with established roles in innate immunity^38–41^. Notably, the TKI-induced IFN response occurs with distinct magnitude and kinetics among the lung cancer cell lines studied, despite similar potencies for growth inhibition. Moreover, a similar variation in the magnitude of the TKI-induced interferon response is observed in human EGFR mutant lung tumor specimens where the IFN response correlates positively with the duration of response of the patients to EGFR inhibitors. The data support a hypothesis that oncogene inhibitor-induced paracrine signaling from lung cancer cells to the immune microenvironment instructs variable degrees of participation by effector immune cells in the observed therapeutic response. Admittedly, our findings do not fully test the hypothesis that recruited immune cells to participate in the overall therapeutic response. To this end, transplantable murine lung cancer cell lines driven by oncogenic EGFR mutations are being developed by our groups to enable mechanistic studies in syngeneic murine hosts. If successful, these model systems will permit approaches to rigorously test the role of the host immune microenvironment in therapeutic response to EGFR-specific TKIs and to define specific secreted factors and immune cell components that may participate.

Recent studies demonstrate similar induction of an IFN response that includes chemokine and cytokines as well as antigen presentation machinery upon treatment with oncogene-targeted drugs in diverse cancer types^42–45^. Treatment of BRAF^V600E^ mutant melanoma with MAPK pathway inhibitors elicited innate immune-related transcriptional responses including upregulation of IFN-responsive genes^46^. Gong et al. observed that TNF levels were induced by EGFR inhibitors, regardless of EGFR mutation status^47^. Similarly, treatment of KRAS mutant lung cancer with MAPK pathway inhibitors induced IFN-regulated genes including CXCL10 and TNF^48^. Furthermore, a recent study by Canon et al. demonstrated that KRAS G12C inhibitor AMG-510 induces a pro-inflammatory microenvironment that is characterized by an increase in IFN signaling, chemokine production, antigen processing, and cytotoxic and NK cell activity^49^. Besides MAPK pathway inhibitors, CDK4/6 inhibitors induce similar responses in breast cancer cells^50^. While it is possible that the observed induction of innate immune responses following treatment with osimertinib is a consequence of growth arrest, our studies indicate equivalent growth inhibition of the EGFR mutant cell lines by this TKI (Supplementary Fig. 1). Moreover, the inhibition of growth-associated gene sets occurs similarly among the cell lines despite significant variation in the magnitude and kinetics of the innate immune response (Fig. 1g and Supplementary Fig. 3). Thus, we suggest that this reprogramming response to osimertinib is not simply a result of inhibited cell growth control mechanisms. Rather, our findings in combination with the studies discussed above support the hypothesis that oncogenes actively suppress IFN pathway activity such that targeted drugs variably unleash this response. Consistent with our results, EGFR-specific TKIs have been found to induce chemokine expression changes that potentially lead to altered CD4+ and CD8+ T-cell content^51^. While we suggest that the TKI-induced IFN response functions in a paracrine fashion to communicate with the immune microenvironment, other studies interpret the response as exerting autocrine effects on the tumor cells yielding bypass signaling and incomplete therapeutic responses. For example, EGFR TKIs were shown to stimulate an IFN response in cell lines with both mutant and wild-type EGFR that functioned in an autocrine mechanism to reduce sensitivity to EGFR TKIs^36^. Similar autocrine signaling roles for TKI-induced Src/IL6/STAT3 and NFκB signaling mediating cell survival have also been demonstrated^31,52^. Thus, the tumor cell autonomous IFN program induction by TKIs and other oncogene-targeted drugs may variably signal in a paracrine fashion as well as through autocrine mechanisms to mediate heterogeneity of individual patient responses within an oncogene-defined set of lung cancer patients.

An important question that remains unanswered by this study is the mechanism(s) accounting for the range of EGFR TKI-induced innate immune responses observed in lung cancer cell lines and primary EGFR mutant lung tumors. At the tumor cell-intrinsic level, the EGFR mutant cell lines exhibited wide-ranging magnitude and kinetics of chemokine/cytokine induction upon treatment with osimertinib (Fig. 2, Supplementary Figs. 3–6) with some exhibiting marked and prolonged responses and other showing little or no induction of these factors. Inspection of mRNA and protein levels for components of the JAK/STAT and IKK/NFκB pathway does not indicate a lack of expression of specific signal pathway elements that may account for this variation in response (data not shown). Epigenetic control of chromatin states that permit TKI-induced regulation of the interferon response seems like a plausible hypothesis to explain the variation in inducibility and warrants a deeper exploration using CHIPseq or ATACseq techniques. Beyond simple TKI-inducibility, our results also reveal that the precise repertoire of the factors secreted by a given cell line is also quite varied (see Fig. 2 and Supplementary Figs. 3–6). This is in contrast to the Induction of MHC class I and II which appeared to be more uniform among the cell lines at the mRNA level (Supplementary Fig. 3). Simplifying the discussion to a single anti-tumorigenic chemokine, CXCL10^11^, and IL6, generally considered to be a pro-tumorigenic chemokine^53^, PC9 cells show balanced induction of both CXCL10 and IL6 protein in response to osimertinib while H1650 cells show a predominant CXCL10 response and HCC827 exhibit a dominant IL6 induction (Fig. 2). The tumor-suppressive cytokine, TGFβ2, was regulated by osimertinib in every cell line except for H1650 cells. Notably, this cytokine is not considered an interferon-inducible gene but is a consensus YAP1-pathway-dependent gene where a recent study demonstrates YAP-mediated transcriptional reprogramming of the apoptotic pathway in lung cancer cells treated with TKIs and MAPK pathway inhibitors^32^. If additional chemokines and cytokines are included in this over-simplified view of the TKI-induced interferon response, a highly varied impact on the immune microenvironment is be predicted to occur if these cell lines reflect primary EGFR mutant lung tumors.

It is worth considering how these findings might be translated clinically to improve the therapeutic response to TKIs in EGFR mutant lung cancer. Inflammation defined by an IFNγ response signature is linked to immune cell infiltration, an important driver of response to immuno-oncology (IO) drugs that target the PD1-PD-L1 immune checkpoint^54–56^. Cancers such as lung cancer, head and neck cancer, and melanoma have exhibited clinical benefit with IO, although patients who are never smokers (i.e., ALK, ROS, and RET) or whose tumors express mutant EGFR showed 0–14% response rates, irrespective of PD-L1 expression levels^57^. Interestingly, Isomoto et al. recently reported that a fraction of EGFR mutant lung cancers sampled before and at TKI-treatment failure (progression) exhibited increased PD-L1 expression and that these tumors maintained higher CD8+ T-cell content^58^. Thus, these findings combined with our studies showing a TKI-induced tumor inflammatory response provides some precedent for combining TKIs with IO. Data from clinical trials testing combinations of TKIs and IO for efficacy in EGFR and ALK-driven lung cancer patents demonstrate modest increases in response rates as compared to single agent IO. These studies are limited in that their main endpoint is the overall response rate, which is already near maximum as TKIs are already highly effective on their own. Based on our present results showing association of the IFNG Hallmark enrichment score with TTP, PFS or overall survival may represent better endpoints. Importantly, these trials have faced challenges resulting from severe toxicities in some patients^59^. The TATTON trial investigated osimertinib in combination with durvalumab in patients with EGFR mutant lung cancer and reported an increase in interstitial lung disease with the combination compared to either drug alone^60^. Furthermore, the phase I CheckMate012 trial investigated erlotinib in combination with nivolumab in EGFR mutant patients and reported multiple incidences of pneumonitis and hepatic toxicities^61,62^. Lastly, the CheckMate370 trial investigated the safety of nivolumab in combination with crizotinib in patients with ALK-positive NSCLC and also reported increases in severe and fatal hepatic toxicities^63^. Collectively, the results from these trials indicate that a combination of TKIs with checkpoint inhibitors may not result in a successful treatment regimen. As the number of IO drugs targeting other immune regulatory mechanisms grows over the next few years, new and less toxic TKI/IO combinations may become available for testing. In Philadelphia chromosome-positive CML, blinatumomab a bispecific anti-CD3/CD19 monoclonal antibody demonstrated efficacy when combined with TKIs such as dasatinib^64^. We propose that a deeper understanding of the basic mechanism of TKI-induced innate immune signaling, especially molecular features that account for the wide response range amongst collections of cancer cell lines and patients, will unveil opportunities for developing combinations of TKIs with novel agents that may enhance TKI-induced IFNγ responses within the tumor or target newly identified immune-related vulnerabilities. Such agents are predicted to increase immune participation in the therapeutic response to TKIs and yield prolonged TTP in patients.

## Methods

### Cell culture

H1650, H1975, HCC827, HCC2935, HCC4006, and PC9 cells were obtained from the University of Colorado Cancer Center Tissue Culture Core and were cultured in RPMI-1640 growth medium supplemented with 10% fetal bovine serum (Sigma, St. Louis, MO, USA) at 37 °C in a humidified 5% CO_2_ incubator. The core laboratory routinely performs DNA fingerprint analysis on all banked cell lines to ensure their authenticity.

### Cell proliferation assay

Cell lines were plated at 100 cells per well in 96-well tissue culture plates and treated in triplicate with test agents. Cell number per well was estimated after 10 days of culture using a CyQUANT Direct Cell Proliferation Assay (Life Technologies, Carlsbad, CA) according to the manufacturer’s instructions.

### Immunoblot analysis

For immunoblot analysis, cells were plated at 250,000 cells per 10-cm dish. After 24 h, cells were treated with 300 nM osimertinib or DMSO control for 3 days. Cells were collected in phosphate-buffered saline, centrifuged (3 min at 3000 rpm), and suspended in MAP Kinase Lysis Buffer. Aliquots of the cell lysates containing 50 µg of protein were submitted to SDS-PAGE on 8% polyacrylamide gels, transferred to nitrocellulose, and immunoblotted. All primary antibodies were used at a dilution of 1:1000 in 3% BSA in 1× TBST. The following antibodies were used: PARP (Cell Signaling Technology 9542S), IFIT1(Cell Signaling Technology 14796S), MX2 (Abcam 22479), STAT1(Cell Signaling Technology 9172S), and ß-actin (Cell Signaling Technology 4967S). The immunoblots were derived from the same experiment processed in parallel.

### RNAseq analysis

Total RNA purified from cultures of DMSO control or AZD9291-treated cultures of PC9, H1650, HCC4006, and H1975 was used to prepare cDNA libraries for sequencing with an Illumina Genome Analyzer. Low-quality sequencing reads were trimmed prior to alignment. Trimmed sequencing reads were mapped against the human genome using the UCSC reference annotation hg19 using HISAT2. Transcripts were assembled against ensemble reference using Cufflinks. Expression is reported as fragments per kilobase transcript per million reads. Data were submitted to applied bias correction and quartile normalization.

### Bioinformatic analysis

GSEA was performed using the log2 ratio of classes metric for ranking genes. The MSigDB v5.2 Hallmarks collection was used as the gene sets and 1000 gene set permutations were performed to determine enriched Hallmark gene sets.

### Patient biopsy collection

Patient samples were collected as part of the Institutional Review Board of the University of Colorado approved protocol (COMIRB #15-2316) and the University of California, San Francisco approved protocol (CC IRB # 13-6512). Lung tumor biopsy specimens were obtained after written informed consent to the IRB-approved protocol #15-2316 entitled “Early Rebiopsy to Identify Mechanisms and Biomarkers of Tumor Cell Survival Following Targeted Therapy in patients with EGFR, ALK, ROS1, or BRAF mutations.” This study permitted enrollment of patients with advanced stage (Stage IIIB/ or IV) EGFR-mutated lung adenocarcinoma who were treatment naive for metastatic disease. Standard of care imaging was done to determine tumor location. Tumors were deemed appropriate for biopsy if they were considered measurable, located at a technically feasible biopsy location, as determined by the study interventional radiologist, and ≥2 cm. Target lesions were biopsied prior to treatment and site matched upon re-biopsy at 2 weeks (±1 week). At each biopsy, 3–5 cores were collected and flash frozen. Histopathologic evaluation of the pre- and post-treatment biopsies was performed within 24–36 h of collection. Each biopsy was sectioned on a cryostat and reviewed at the time of frozen section by a board-certified pathologist. The presence of tumor, percentage of necrosis, and overall estimated tumor/stromal cellularity were recorded for each biopsy core. The H&E slides were barcoded and archived. The highest quality core(s) were selected for RNA extraction with a goal of obtaining at least 500 tumor cells to facilitate optimal RNA sequencing.

### Quantitative real-time PCR (qRT-PCR)

Five hundred thousand cells were seeded in 10-cm plates and allowed to attach. After, 24 h, cells were treated with DMSO or 300 nM osimertinib for 4 h to 21 days. Cells were collected in 600 µL RNA Lysis Buffer, total RNA was purified from cells using Quick-RNA MiniPrep kits (Zymo Research, Irvine, CA) and aliquots (5 µg) were reverse transcribed in a volume of 20 µL using Maxima First Strand cDNA Synthesis Kit (Thermo Scientific, Pittsburgh, PA). Aliquots (2 µL) of a 1:5 dilution of the reverse transcription reactions were submitted to quantitative RT-PCR in 10 µL reactions with SYBR Select Master Mix for CFX (Thermo Fisher Scientific) with the primers listed in Supplementary Table S1 using a CFX Connect Real-Time PCR Detection System (BioRad, Hercules, CA). The real-time PCR amplification products from initial experiments were resolved by electrophoresis on 5% polyacrylamide gels to verify that the primer pairs yielded a single amplicon of the predicted sizes. GAPDH mRNA levels were measured as a housekeeper gene for normalization of the different mRNA expression values, and the data are presented as “Relative Expression”.

### CXCL10 and IL6 ELISA

Cells were seeded in 10 cm dishes and 24-h later, the cells were treated with DMSO vehicle control, 300 nM osimertinib, 500 nM ruxolitinib, 500 nM IKK16 or the combinations. The media was collected and assayed for CXCL10 or IL6 according to the manufacturer’s instructions (Quantikine human CXCL10/IP-10 ELISA kit; R&D Systems, Minneapolis, MN). The concentration of CXCL10 or IL6 in the media was normalized to the total cellular protein per dish and the data are presented as pg/µg.

### Luminex assay

Cells were seeded in 10-cm dishes and 24-h later, the cells were treated with DMSO vehicle control or 300 nM osimertinib. The media was collected and assayed for the CXCL6, CXCL12, OPN, CCL20, CCL22, IL1a, IL4, IGFBP3, CSF1, CSF2, CXCL10, IL6, CCL5, and CXCL1 analytes according to the manufacturer’s instructions (Luminex kit; R&D Systems, Minneapolis, MN). The concentration of analyte in the media was normalized to the total cellular protein per dish and the data are presented as pg/µg.

### Dominant-negative IkB

The mutant IκB retroviral plasmid pQCXIP-mIκBα and pQCXIP empty vector control was kindly provided by Dr. Rebecca Schweppe^65^ (University of Colorado Anschutz Medical Campus). Both constructs were packaged in 293T cells (obtained from the University of Colorado Cancer Center Tissue Culture Core) with retroviral packaging component vectors pSV- Ψ^−^ -env-MLV and pSV- Ψ^−^-A-MLV. The retroviruses released into the medium were filtered using a 0.45-μm filter and used to transduce PC9 cells in 10-cm plates at 500,000 cells/dish. Transduced cells were selected with puromycin (1 μg/ml) for 8 days to generate stable cell lines expressing dnIκB or pQCXIP.

### Immunofluorescence

Immunofluorescence was performed on 5-micron sections cut fresh from frozen tissue embedded in optimal cutting temperature and subjected to antibody staining within 4 weeks after sectioning. The sections were immediately imaged, and image analysis was employed to quantify positive cells. For quantification of T cells, the total number of CD3^+^ T cells was counted from five independent fields per tumor. Due to tumor size limitations, five fields were sufficient to capture the entire tumor for each sectioned biopsy. ImageJ software version 1.8.0 was used to visualize images and perform data analysis.

### PC9 and H1650 xenografts

PC9 and H1650 cells were suspended in 50% Matrigel/phosphate-buffered saline at 10 million cells per mL and 1 million cells were injected subcutaneously in both flanks of female nu/nu mice. When at least one of the tumors reached a volume of 100 mm^3^, the mice were randomized into treatment groups of diluent control (1% Polysorbate 80), AZD9291 (5 mg/kg). Drugs were delivered daily by oral gavage and weight was monitored weekly for signs of morbidity. Tumor volumes were measured using calipers. The study protocol was approved by the University of Colorado Anschutz Office of Laboratory Animal Resources. All animal studies complied with relevant ethical regulations.

### Reporting summary

Further information on research design is available in the Nature Research Reporting Summary linked to this article.

## Supplementary information

Supplementary Data 1

Supplementary Data 2

Supplementary Data 3

Reporting Summary

Supplementary Information

## Data Availability

The data generated and analyzed during this study are described in the following data record: 10.6084/m9.figshare.14345930^66^. The RNA sequencing data are openly available in the Gene Expression Omnibus (GEO) repository via the following accession: https://identifiers.org/geo:GSE165019^67^. The GEO data will be made publicly available within one year of the publication of the article. Other data generated and analyzed during the related study are in the following Prism and Excel files: Figure 2.pzfx, Figure 3.pzfx, Figure 5.pzfx, SF1.pzfx, SF3.pzfx, SF.4pzfx, S5.pzfx, S6.pzfx, SuppTable1.xslx, SuppTable2.xlsx, SuppTable3.xslx. These files are not openly available, but can be made available upon written request to the corresponding authors. Human subject data (de-identified and anonymised) can be made available upon request to the corresponding authors.”
