# Supplementary material for: A tyrosine kinase inhibitor-induced interferon response positively associates with clinical response in EGFR-mutant lung cancer
Source: NPJ Precis Oncol. 2021 May 17;5:41. doi: 10.1038/s41698-021-00181-4 (PMC8129124; doi:10.1038/s41698-021-00181-4)
Supplement: Supplementary file 4 — Reporting Summary [file 41698_2021_181_MOESM4_ESM.pdf]

nature research

Corresponding author(s): Lynn Heasley, Robert Doebele

Last updated by author(s): Jan 28, 2021

Reporting Summary

Nature Research wishes to improve the reproducibility of the work that we publish. This form provides structure for consistency and transparency in reporting. For further information on Nature Research policies, see our Editorial Policies and the Editorial Policy Checklist.

Statistics

For all statistical analyses, confirm that the following items are present in the figure legend, table legend, main text, or Methods section.

n/a	Confirmed	
☐	☒	The exact sample size (*n*) for each experimental group/condition, given as a discrete number and unit of measurement
☐	☒	A statement on whether measurements were taken from distinct samples or whether the same sample was measured repeatedly
☐	☒	The statistical test(s) used AND whether they are one- or two-sided<br>*Only common tests should be described solely by name; describe more complex techniques in the Methods section.*
☐	☒	A description of all covariates tested
☐	☒	A description of any assumptions or corrections, such as tests of normality and adjustment for multiple comparisons
☐	☒	A full description of the statistical parameters including central tendency (e.g. means) or other basic estimates (e.g. regression coefficient) AND variation (e.g. standard deviation) or associated estimates of uncertainty (e.g. confidence intervals)
☐	☒	For null hypothesis testing, the test statistic (e.g. *F*, *t*, *r*) with confidence intervals, effect sizes, degrees of freedom and *P* value noted<br>*Give P values as exact values whenever suitable.*
☒	☐	For Bayesian analysis, information on the choice of priors and Markov chain Monte Carlo settings
☒	☐	For hierarchical and complex designs, identification of the appropriate level for tests and full reporting of outcomes
☒	☐	Estimates of effect sizes (e.g. Cohen's *d*, Pearson's *r*), indicating how they were calculated

Our web collection on statistics for biologists contains articles on many of the points above.

Software and code

Policy information about availability of computer code

Data collection	For RNA-seq analysis, RNA was submitted to the University of Colorado Bioinformatics core where library prep was generated and RNA was sequenced on the NovaSeq 4000 to generate 2x151 reads. Fastq files were quality checked with FastQC, illumina adapters trimmed with bbduk, and single or paired reads were mapped to the human UCSC hg38 BWA genome with samtools 1.11 bwa-mem. Output sam files were converted to bam format and sorted with samtools 1.11. Bam files were processed with picard tools 1.119 (AddOrReplaceReadGroups, ReorderSam, and MarkDuplicates). GATK 3.3
Data analysis	For RNA-seq analysis, RNA was submitted to the University of Colorado Bioinformatics core where library prep was generated and RNA was sequenced on the NovaSeq 4000 to generate 2x151 reads. Fastq files were quality checked with FastQC, illumina adapters trimmed with bbduk, and single or paired reads were mapped to the human UCSC hg38 BWA genome with samtools 1.11 bwa-mem. Output sam files were converted to bam format and sorted with samtools 1.11. Bam files were processed with picard tools 1.119 (AddOrReplaceReadGroups, ReorderSam, and MarkDuplicates). GATK 3.3

For manuscripts utilizing custom algorithms or software that are central to the research but not yet described in published literature, software must be made available to editors and reviewers. We strongly encourage code deposition in a community repository (e.g. GitHub). See the Nature Research guidelines for submitting code & software for further information.

Data

Policy information about availability of data

All manuscripts must include a data availability statement. This statement should provide the following information, where applicable:

- Accession codes, unique identifiers, or web links for publicly available datasets
- A list of figures that have associated raw data
- A description of any restrictions on data availability

All data sets involving research presented in this manuscript regarding animals and basic science will be shared upon written request. In addition, RNAseq files are available via GEO Datasets at PubMed.gov (GEO accession number GSE165019). Publications from this research will be made available to the public through the National Library of Medicine PubMed Central website within on year after the date of publication (guidance is provided on the ORD website). Regarding human subject data, a de-identified, anonymized dataset will be created and shared.

Field-specific reporting

Please select the one below that is the best fit for your research. If you are not sure, read the appropriate sections before making your selection.

☒ Life sciences ☐ Behavioural & social sciences ☐ Ecological, evolutionary & environmental sciences

For a reference copy of the document with all sections, see nature.com/documents/nr-reporting-summary-flat.pdf

Life sciences study design

All studies must disclose on these points even when the disclosure is negative.

Sample size	For in vitro studies with EGFR mutant cell lines, all cell lines that were readily available were studied. For analysis of human tumor biopsies, All available biopsies from EGFR mutant lung cancer patients that had been accrued were investigated. There was no a priori sample size selected.
Data exclusions	no data were excluded
Replication	Three independent experiments were performed when possible to assess experiment to experiment replication with in vitro studies.
Randomization	All EGFR mutant lung cancer patients were treated with a TKI. The study was not designed to test efficacy of a particular drug.
Blinding	Blinding was not performed as it was not applicable to the studies performed.

Reporting for specific materials, systems and methods

We require information from authors about some types of materials, experimental systems and methods used in many studies. Here, indicate whether each material, system or method listed is relevant to your study. If you are not sure if a list item applies to your research, read the appropriate section before selecting a response.

Materials & experimental systems

n/a	Involved in the study	
☐	☒	Antibodies
☐	☒	Eukaryotic cell lines
☒	☐	Palaeontology and archaeology
☐	☒	Animals and other organisms
☐	☒	Human research participants
☐	☒	Clinical data
☒	☐	Dual use research of concern

Methods

n/a	Involved in the study	
☒	☐	ChIP-seq
☒	☐	Flow cytometry
☒	☐	MRI-based neuroimaging

Antibodies

Antibodies used	Antibodies from Cell Signaling Technologies: B-actin cat# 4967S Lot 12, STAT1 cat# 9172S Lot 25, IFIT1 Cat #14796S Lot 1, PARP cat#9542S Lot 14, Anti-rabbig IgG, HRP-linked Cat# 7074S lot 29. Abcam antibodies: MX2 cat# 22479 Lot GR3198852-3. ThermoSci antibodies: CD3 Cat#MA5-14524 rabbit anti mouse CD3epsilon clone: SP7. VectaCell DAPI Cat#CB-2000
Validation	The antibodies used have been validated by the commercial source. In addition, care was taken to ensure that the immunoblotted proteins migrated with the appropriate molecular marker.

Eukaryotic cell lines

Policy information about cell lines

Cell line source(s)	All cell lines were obtained from the University of Colorado Cancer Center Cell Technologies Core
Authentication	The cell lines are routinely submitted to STR/finger print analysis by the Cell Technologies Core.
Mycoplasma contamination	The cell lines used in the studies were negative for mycoplasma.
Commonly misidentified lines (See ICLAC register)	No commonly misidentified cell lines were used.

Animals and other organisms

Policy information about studies involving animals; ARRIVE guidelines recommended for reporting animal research

Laboratory animals	nu nu, female, 6 weeks old
Wild animals	study did not involve wild animals
Field-collected samples	study did not require field collected samples
Ethics oversight	University of Colorado Anschutz Office of Laboratory Animal Resources provided ethics guidance on the study protocol.

Note that full information on the approval of the study protocol must also be provided in the manuscript.

Human research participants

Policy information about studies involving human research participants

Population characteristics	The tumor biopsies were obtained from lung cancer patients whose tumors were positive for an oncogenic EGFR mutation. There was no other selection for population except for their willingness to consent to a repeat, on-treatment biopsy.
Recruitment	The patients were recruited due to diagnosis for EGFR mutant lung cancer in the Thoracic Oncology clinics at the University of Colorado or University of California San Francisco Hospitals.
Ethics oversight	The clinical protocol for obtaining research biopsies was approved by the Colorado Multiple Institutional Review Board.

Note that full information on the approval of the study protocol must also be provided in the manuscript.

Clinical data

Policy information about clinical studies

All manuscripts should comply with the ICMJE guidelines for publication of clinical research and a completed CONSORT checklist must be included with all submissions.

Clinical trial registration	NCT03042221
Study protocol	The full protocol can be obtained from the Colorado Multiple Institutional Review Board.
Data collection	The clinical data were collected in the Thoracic Oncology clinics at the University of Colorado Hospital and University of California San Francisco from 2016 through 2019. The data were stored in secure databases and after being stripped of identifiers, provided to the investigators for correlation analysis.
Outcomes	Time to progression (TTP), was measured by the Thoracic Oncology teams as the time point at which tumor re-growth had occurred.
