# Supplementary material for: A tyrosine kinase inhibitor-induced interferon response positively associates with clinical response in EGFR-mutant lung cancer
Source: NPJ Precis Oncol. 2021 May 17;5:41. doi: 10.1038/s41698-021-00181-4 (PMC8129124; doi:10.1038/s41698-021-00181-4)

Supplementary Information for

A Tyrosine Kinase Inhibitor- Induced Interferon Response Positively Associates with Clinical Response in EGFR-Mutant Lung Cancer

Natalia J. Gurule[1], Caroline E. McCoach[4], Trista K. Hinz[1], Daniel T. Merrick[3], Adriaan Van Bokhoven[3], Jihye Kim[2], Tejas Patil[2], Jacob Calhoun[1], Raphael A. Nemenoff[2], Aik Choon Tan[5], Robert C. Doebele[2*] and Lynn E. Heasley[1,6*]

Departments of Craniofacial Biology[1], Medicine[2], and Pathology[3], University of Colorado Anschutz Medical Campus, Aurora, Colorado

[6]Eastern Colorado VA Healthcare System, Rocky Mountain Regional VA Medical Center, Aurora, Colorado

[4]Department of Medicine and Helen Diller Family Comprehensive Cancer Center, University of California, San Francisco, California

[5]Moffitt Cancer Center, Tampa, Florida

A

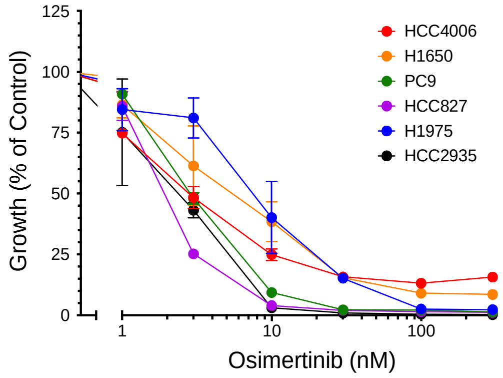

B

Cell Line	Mutations	Osimertinib IC_{50} (nM)
HCC4006	EGFR del19, TP53	3
H1650	EGFR del19, TP53	6
H1975	EGFR L858R, T790M, TP53	9
PC9	EGFR del19, TP53	3
HCC827	EGFR del19, TP53	2
HCC2935	EGFR del19, TP53	2

C

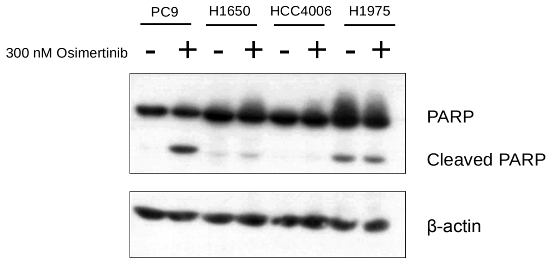

Supplementary Figure 1. Osimertinib sensitivity of the EGFR mutant lung cancer cell lines. A. The EGFR mutant lung cancer cell lines were tested for sensitivity to osimertinib using 96-well clonogenic growth assays described in the Materials and Methods. Dose-response curves showing mean and SEM of triplicate measurements are shown and representative of two independent experiments. **B.** Features of the human EGFR mutant lung cancer cell lines and the osimertinib IC_{50} values are tabulated. **C.** PC9, H1650, HCC4006 and H1975 cells were treated *in vitro* with 300 nM osimertinib for 3 days. PARP and cleaved PARP were measured by immunoblotting as described in the Materials and Methods. The filter was stripped and re-probed for β-actin. The data are from the same experiment and processed in parallel.

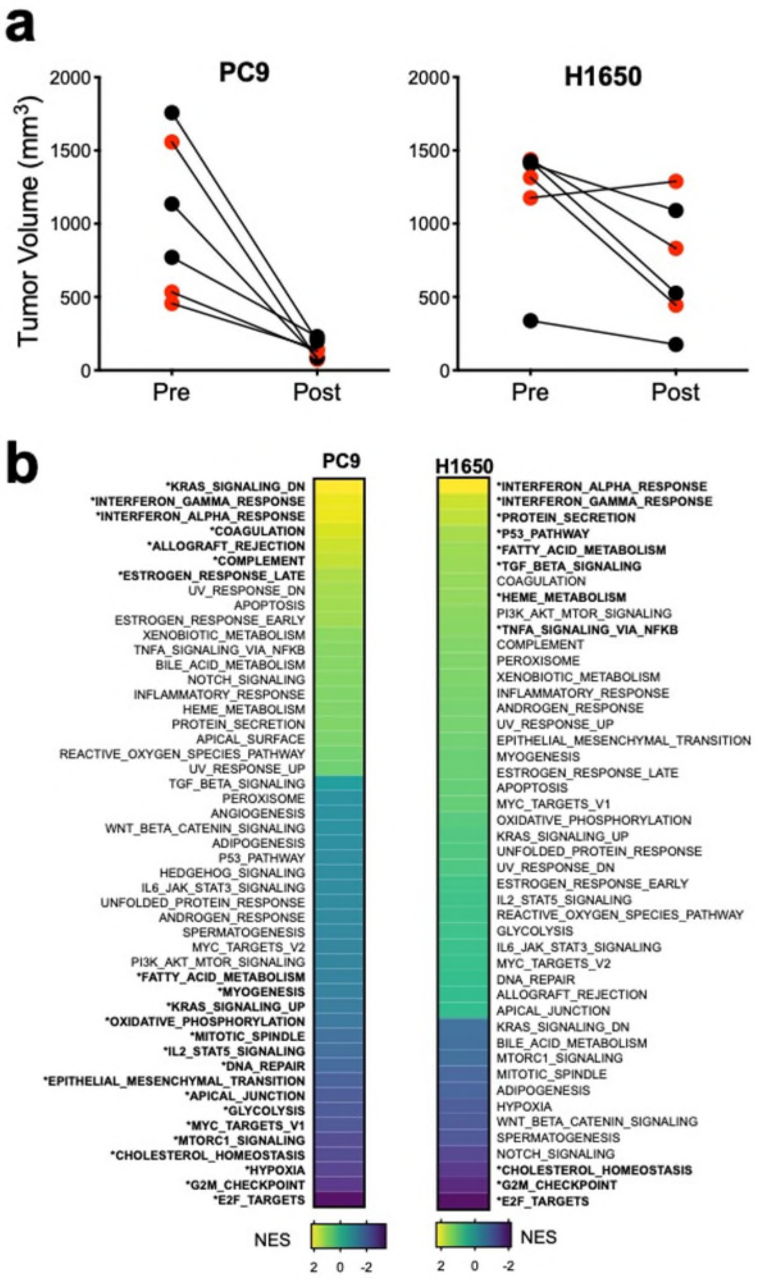

Supplementary Figure 2 In vivo response of H1650 and PC8 xenografts to osimertinib treatment. H1650 and PC9 cells were implanted in the flanks of nu/nu mice and tumors were allowed to establish for ~5-7 weeks until the xenografts were 500 – 1,500 mm^3. The tumor-bearing mice were treated by daily oral gavage with 5 mg/kg osimertinib or diluent control for 2

weeks and tumor volumes were measured with calipers twice weekly. Following the 2-week treatment, the tumors were harvested, RNA was purified and submitted to RNAseq. **A.** Individual flank tumor response to osimertinib. The tumors indicated in red were submitted to RNAseq. **B.** Gene Set Enrichment Analysis was performed using the Hallmarks MSigDB gene sets and Normalized Enrichment Scores (NES) are indicated for the osimertinib-treated tumors relative to the diluent control treated (n=3). The Hallmark Pathways indicated in bold are statistically significant as assessed by nominal $p<0.05$.

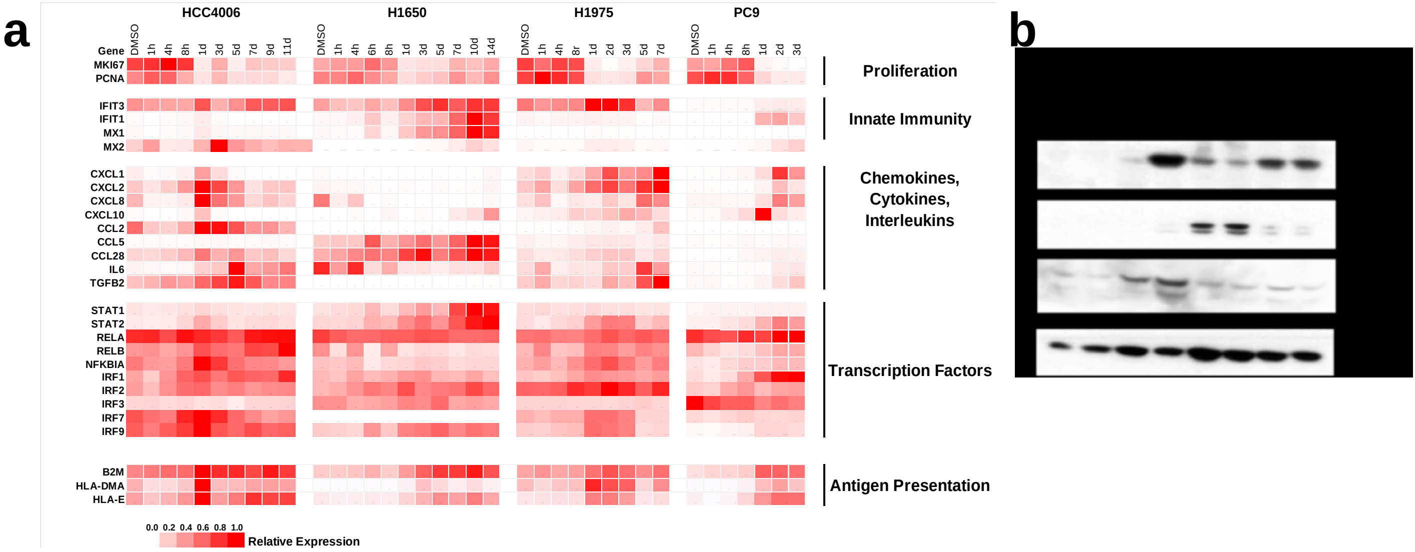

Supplementary Figure 3. Variation in the magnitude and kinetics of induction of selected IFN-responsive genes by osimertinib. A, The RNAseq expression values (FPKM) for the genes shown were normalized (0-1) across the 4 cell lines and presented with the time of osimertinib treatment. A value of 1 represents the maximum FPKM value for that gene among the 4 cell lines. Genes with indicated functions in the innate immune response are grouped in the heatmap representation. **B,** Cell-free extracts from PC9, H1650, HCC4006 and H1975 cells treated with and without osimertinib for 2, 10, 2 and 2 days, respectively were submitted to immunoblot analysis for IFIT1, MX2 and STAT1 protein levels. The filters were stripped and re-probed for β-actin as a loading control. The data are from the same experiment and processed in parallel.

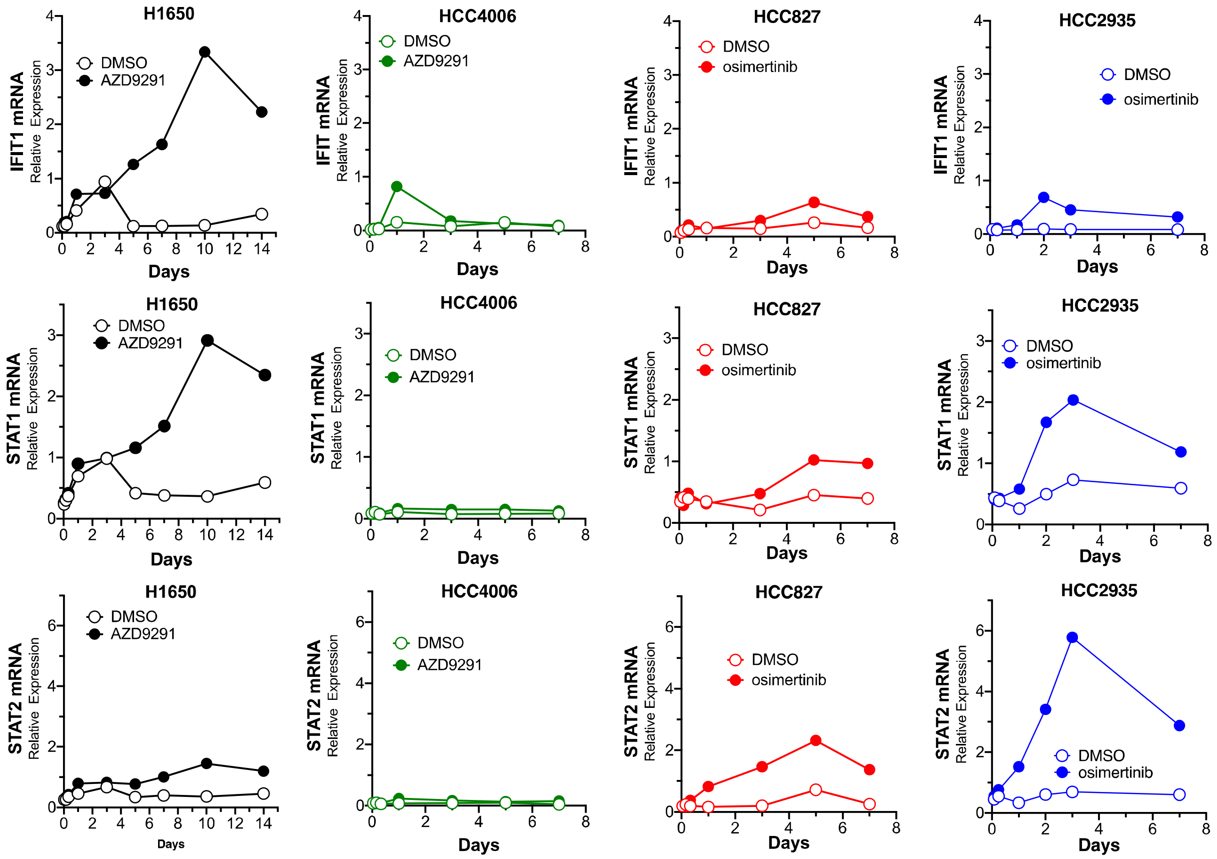

Supplementary Figure 4. Validation of IFIT1, STAT1 and STAT2 mRNA regulation by osimertinib treatment by quantitative RT-PCR. H1650, HCC4006, HCC827 and HCC2935 cells were treated *in vitro* with 300nM osimertinib or DMSO control for the indicated times. RNA was isolated and submitted to qRT-PCR to measure STAT1, STAT2, and IFIT1 mRNA as described in the Materials and Methods. The data were normalized to GAPDH mRNA levels measured in the same samples and presented as relative expression. The data are from 1 distinct sample per time point.

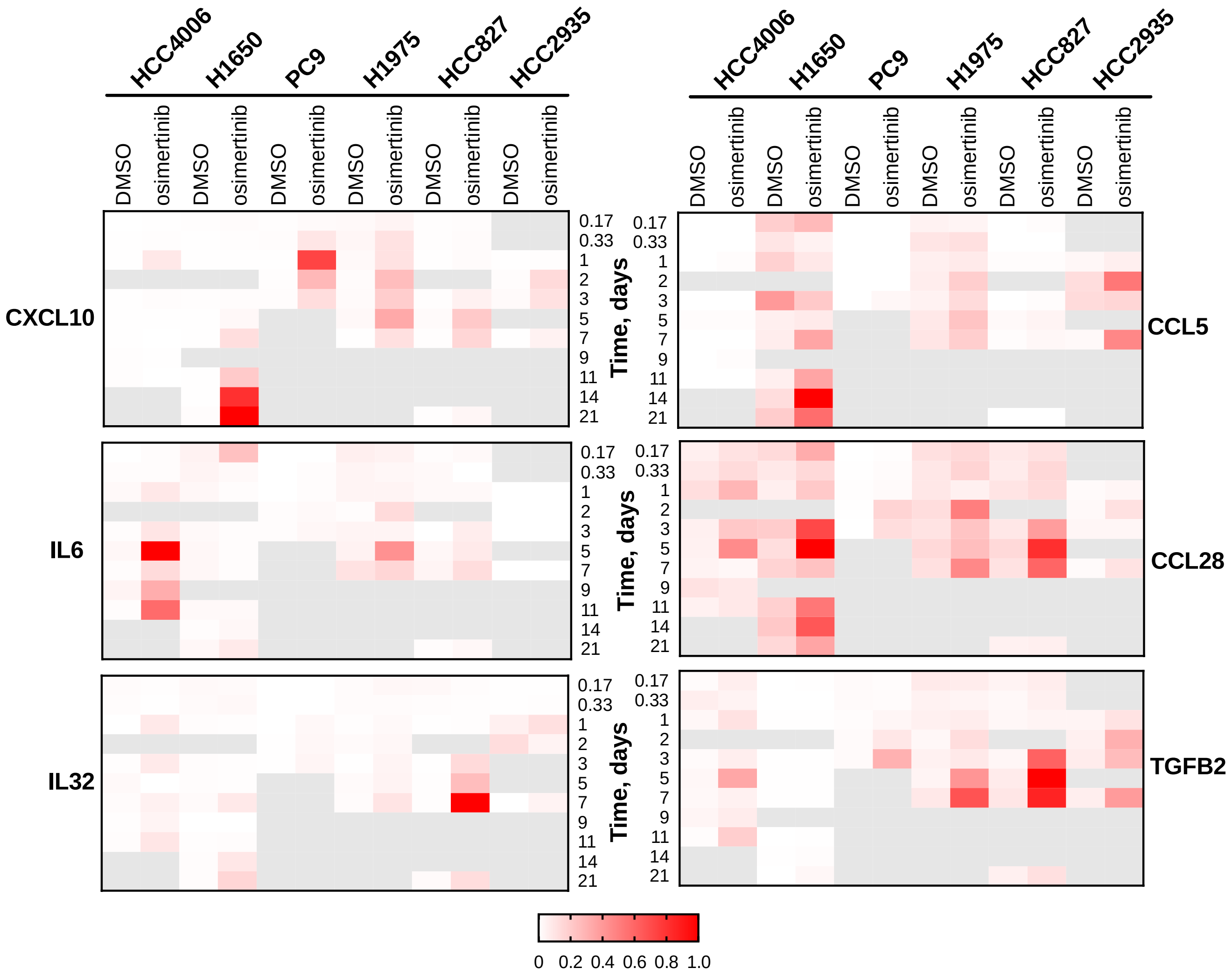

Supplementary Figure 5. Measurement of diverse chemokine and cytokine mRNA expression changes induced by osimertinib treatment in EGFR mutant lung cancer cell lines. HCC4006, H1650, PC9, H1975, HCC827, and HCC2935 cells were treated *in vitro* with 300nM osimertinib or DMSO control for the indicated times. RNA was isolated and submitted to qRT-PCR for CXCL10, IL6, IL32, CCL5, CCL28, and TGFβ2 mRNA. The measurements were normalized to GAPDH and each gene was internally normalized (0-1) across the six cell lines such that a value of 1 represents the maximum relative expression for that gene. Cells filled with

light grey indicate no values were obtained in a particular cell line at that time point. The data are from 1 distinct sample per time point.

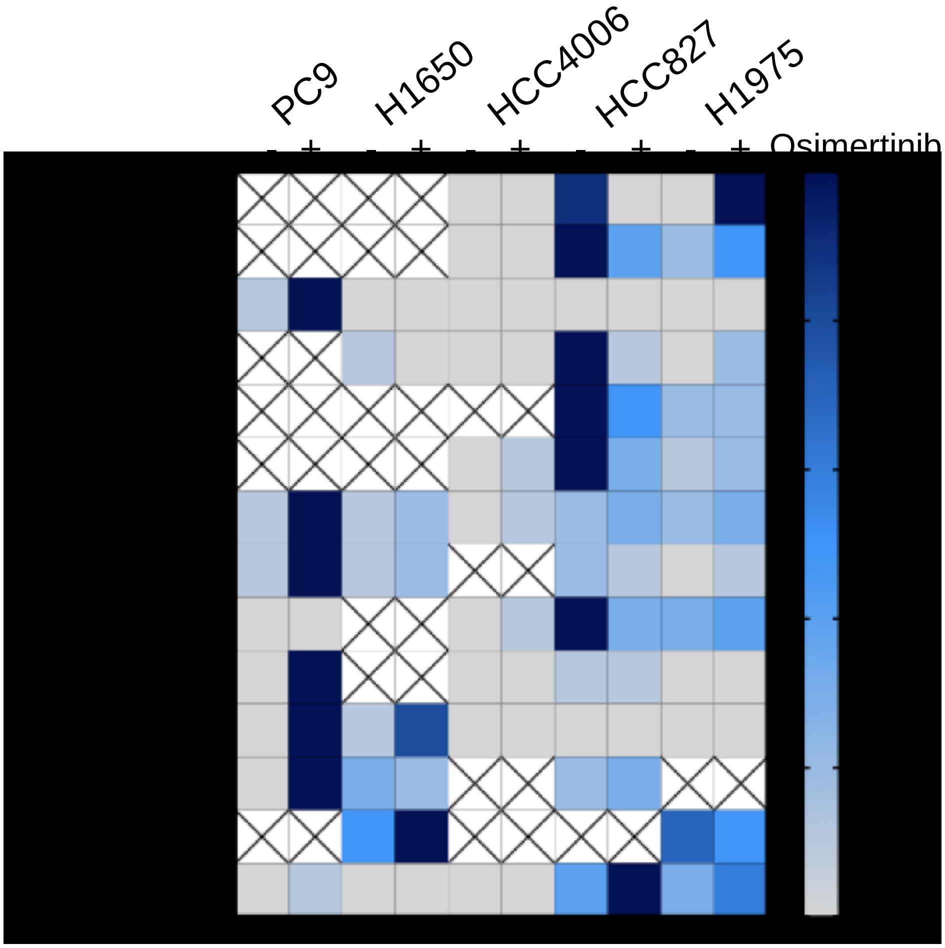

Supplementary Figure 6. Luminex-based analysis of secreted signaling molecules in conditioned medium from osimertinib-treated lung cancer cell lines. HCC4006, H1650, PC9, H1975, and HCC827 cells were treated *in vitro* with 300 nM osimertinib or DMSO control. Treatment time points were as follows, HCC4006, 3 days; H1650, 7 days; PC9, 3 days; H1975, 7 days; HCC827, 5 days. Media was collected and submitted to a Luminex assay to measure protein secretion of the indicated analytes.1 distinct sample per each condition was measured and the measurements were normalized to total cellular protein and presented as pg/μg.

Supplementary Table 1. Primers used for quantitative RT-PCR. The oligonucleotide primers used for the experiments in Supplementary Figures S3 and S4 are tabulated.

Supplementary Table 2. Gene Set Enrichment Analysis of patient-derived biopsy RNAseq data. GSEA was performed on the RNAseq data to identify Hallmark Pathways enriched in the rebiopsy specimens (Rebiopsy vs. Baseline) as well as pathways enriched in the baseline sample (Baseline vs. Rebiopsy). The enrichment score (ES), normalized enrichment score (NES), nominal p-value (NOM p-val), false discovery rate q-value (FDR q-val) and the familywise error rate p-value (FWER p-val) are tabulated for each Hallmark Pathway and each baseline-rebiopsy comparison. The specific Hallmark pathways are ranked from left to right by the average NES score among the 8 biopsy pairs.

Supplementary Table 3. Pearson correlation analysis of GSEA Hallmark pathway enrichment scores from patient paired biopsy RNAseq data and time to treatment progression. The enrichment scores for the 50 Hallmark pathways enriched in the rebiopsy specimens and the time to progression measured in the 8 patients were submitted to Pearson correlation analysis. The Pearson r and p-values are tabulated as well as the number of ES values that were used in the analysis. The resulting data were filtered to only include those Hallmark pathways that were identified in at least 7 of the 8 biopsy pairs and then ranked by p-value. Only the IFNG Hallmark pathway was significantly associated with TTP, although the IL6-

JAK-STAT pathway, Allograft Rejection and IFNA pathways had p-values that trended towards significance.

Uncropped Immunoblots from Supplemental Figure 1C and Supplemental Figure 3B

Uncropped western blot for Supplemental Figure 1C

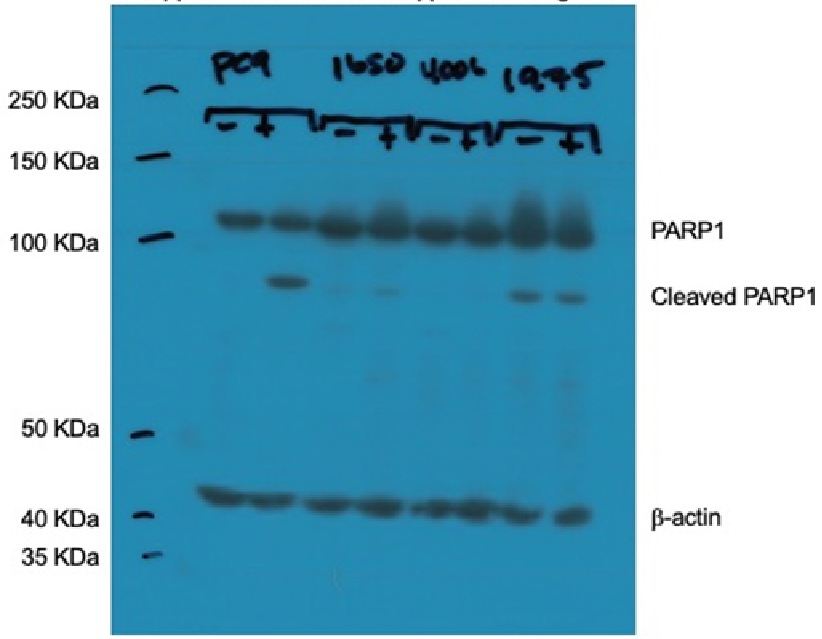

Uncropped western blot for Supplemental Figure 3B

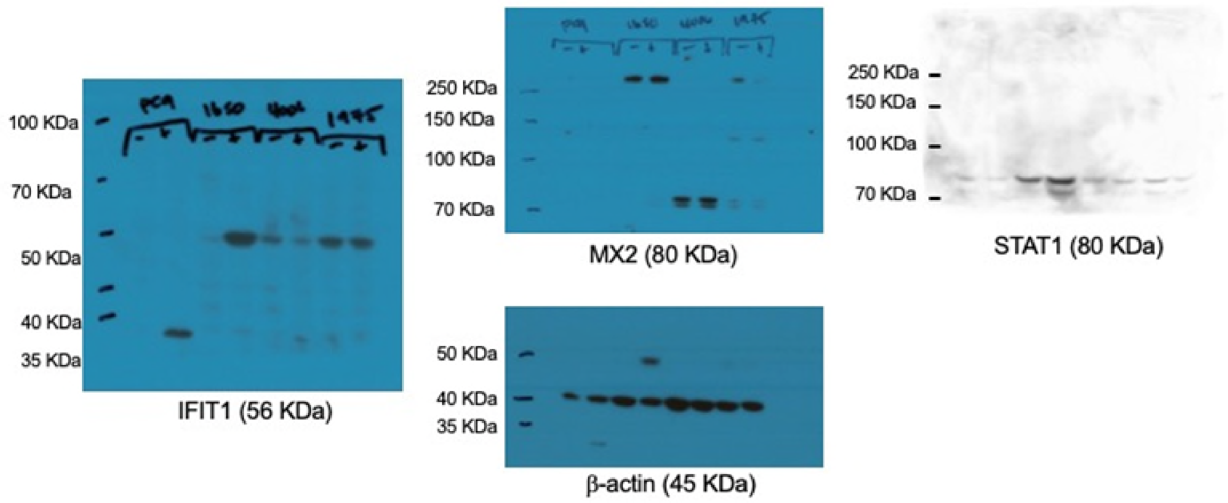

Supplement: Supplementary file 5 — Supplementary Information [file 41698_2021_181_MOESM5_ESM.pdf]
